# Physiological synchrony among human fishers during collective hunting with wild dolphins

**DOI:** 10.1098/rsbl.2025.0421

**Published:** 2025-10-29

**Authors:** Hanja B. Brandl, João V. S. Valle-Pereira, Jens C. Pruessner, Alexandre M. S. Machado, Fábio G. Daura-Jorge, Mauricio Cantor, Damien R. Farine

**Affiliations:** ^1^ Centre for the Advanced Study of Collective Behaviour, Universität Konstanz, Konstanz, Germany; ^2^ Department of Collective Behaviour, Max-Planck-Institut für Verhaltensbiologie, Konstanz, Germany; ^3^ Department of Fisheries, Wildlife and Conservation Sciences, Marine Mammal Institute, Oregon State University, Newport, OR, USA; ^4^ Department of Psychology, Universitat Konstanz, Konstanz, Baden-Württemberg, Germany; ^5^ Departamento de Ecologia e Zoologia, Universidade Federal de Santa Catarina, Florianopolis, Santa Catarina, Brazil; ^6^ Department of Evolutionary Biology and Environmental Studies, University of Zurich, Zurich, Switzerland; ^7^ Division of Ecology and Evolution, Research School of Biology, Australian National University, Canberra, Australian Capital Territory, Australia

**Keywords:** arousal, collective behaviour, heart rate variability, human–wildlife, cooperation, physiological alignment, predator–prey interactions, social hunting

## Abstract

Predator physiology is often overlooked in predator–prey interactions, despite its potential to significantly influence hunting dynamics and social cooperation among predators. We address this gap by investigating how physiological alignment relates to group dynamics and hunting performance in a unique interspecies mutualism: artisanal net-casting fishers who target fish with assistance from wild dolphins. We monitored 24 fishers using high-resolution chest-belt sensors, recording continuous electrocardiograms (ECG) and GPS positions. We then calculated interpersonal heart rate variability (HRV) synchrony in low (LF) and high frequency (HF) bands while tracking changes in foraging and social contexts. In both LF and HF HRV domains, positive social factors increased HRV synchrony among fishers, especially those with established cooperative bonds, whereas distance between fishers decreased HRV synchrony. External factors—the presence and activity of dolphins—had no measurable impact on HRV synchrony. We also found a negative association between group-level hunting success and HRV synchrony in the LF domain, which is influenced by both sympathetic and parasympathetic activity. By demonstrating the role of physiological synchrony during collective hunting in humans—driven by social factors and with direct implications for hunting outcomes—our study advances the current understanding of the eco-physiological dynamics of social predators in predator–prey systems.

## 1. Introduction

Predation forms the backbone of food webs, often driving cascading effects on ecosystem functioning [1,2]. Unsurprisingly, predator–prey interactions have long been central in ecological research. While collective responses [3] and physiological mechanisms in prey species [4,5] are well-studied, both aspects remain largely neglected in group hunting contexts (but see [6])—limiting our understanding of how physiological processes shape hunting dynamics. Insights from other fields, such as sports [7,8] and task performance [9,10], suggest that physiological processes can shape group outcomes, but little is known about their role in predators that forage cooperatively. In these systems, physiology may affect not only individual performance, but also social relationships and collective abilities in fitness-critical tasks [11,12].

Synchrony—temporal alignment of actions, emotions or physiological states between individuals—is a key mechanism shaping group coordination and cooperation [13–15]. Mirroring behaviours like movements, postures and gestures is common in both human [16,17] and non-human [18–20] social interactions. Interpersonal synchrony strengthens social bonds by increasing cohesion [15,17,21], facilitating information flow and trust [22,23], and ultimately improving collective performance [13,24,25]. Emerging evidence suggests that synchrony in the autonomic nervous system (ANS) activity, such as in heart rate (HR) and heart rate variability (HRV)—the fluctuation in time between heartbeats—can be highly relevant in these contexts [13,26,27]. The ANS regulates involuntary physiological responses, including HR and breathing, and modulates arousal, emotional states, and stress during social interactions [27]. Measuring ANS synchrony across individuals therefore provides insights into the physiological basis of bonding and cooperation. Specifically, HRV reflects the interplay between sympathetic (arousal) and parasympathetic (‘rest and digest’) activity [28], making it a useful indicator of the ANS’ adaptability to environmental conditions [29,30], resilience to stress [31,32] and social responsiveness [26,33].

In social predation, individuals benefit from hunting together through coordination and cooperation [34,35]. Artisanal net-casting fishers in southern Brazil exemplify a human social predation tactic. These fishers coordinate their casts with one-another as part of their synchronized responses to mutualistic interactions with wild Lahille’s bottlenose dolphins (*Tursiops truncatus gephyreus*). These dolphins herd migratory mullet (*Mugil liza*) schools towards shallow estuarine waters where groups of fishers stand, waiting for specific dolphin cues—sudden dives with arched backs or head and tail slaps—that indicate the fish location and optimal net-casting moments [36,37]. While fishers compete to catch fish, they also benefit by coordinating their actions with others [38]. As a result, many cooperate by sharing prime fishing spots, casting nets side-by-side, sharing daily catch and sales, and division of labour where one fisher manages the catch while another sells it [39]. This intricate system—combining intra-group cooperation and competition during interspecies mutualism—offers a rare and accessible natural model for exploring complex links between physiology, group dynamics, and cooperation during group hunting.

Here, we investigate physiological synchrony among fishers during both cooperative intraspecific interactions and mutualistic fishing with dolphins, to explore eco-physiological dynamics in a predator–prey system. HRV synchrony provides insight into shared physiological responses to collective experiences and offers a powerful tool to identify the social and environmental factors that promote or disrupt alignment among fishers, and its contribution to fishing success. We hypothesize that physical proximity and cooperative bonds enhance HRV synchrony among fishers, and that dolphins’ presence and behaviour further influence synchronization by providing salient external cues that help coordinate fishers’ attention and actions. We also predict that higher HRV synchrony improves collective hunting success by increasing cohesion and coordination [15,21,40].

## 2. Methods

### (a) Study system

**Figure 1.**
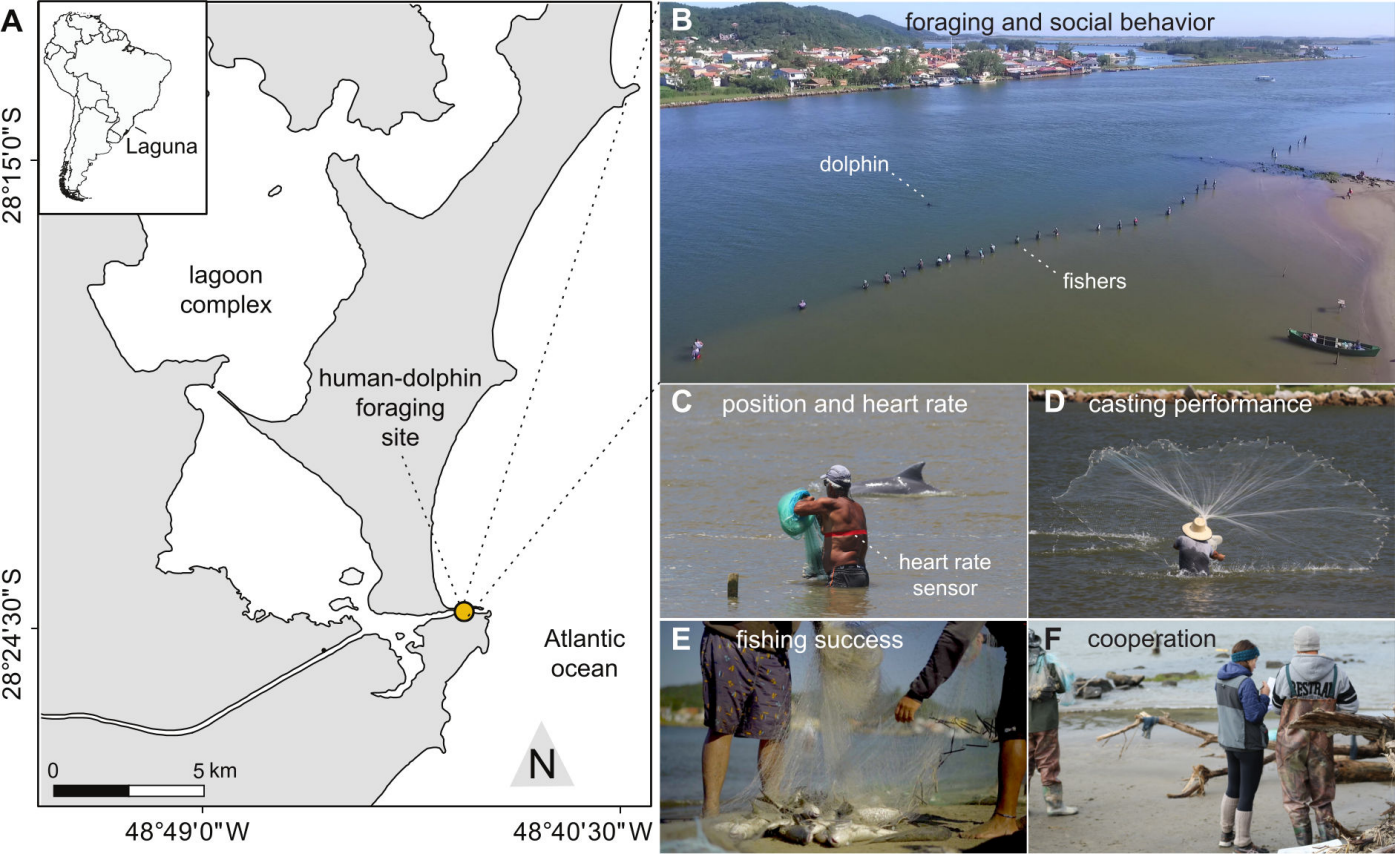
Cooperative artisanal net-casting fishers hunt with dolphin assistance. (A)*Tesoura* beach is located in a canal connecting an estuarine system to the Atlantic Ocean in Laguna, Brazil. (B) Fishers stand in the water along the canal edge, waiting for cues from dolphins indicating the presence of mullets and the optimal time and place to cast nets. We continuously recorded (C) fishers’ group composition and dolphin presence, (D) the exact timing of net-casting events to assess social and foraging contexts, (E) successful catches, quantified as the number of mullet caught per net-cast and (F) fisher–fisher cooperation, expressed through sharing fishing spots, sales or catch processing, was recorded using semi-structured interviews. Photos: (B) A. M. S. Machado, (C,F) M. Cantor, (D) F. G. Daura-Jorge, (E) Mysticeta Research Co.; reproduced with permission.

In southern Brazil, traditional net-casting fishers stand by lagoon margins, awaiting cues from wild Lahille’s bottlenose dolphins herding mullet schools, which indicate when and where to cast their nets (figure 1A,B) [37]. Here, fishers can compete or collaborate with other fishers. Because fishing spots are limited along the approximately 100 m margin, fishers self-organize their fishing activities following an informal rule system: spots are allocated on a first-come, first-served basis; and fishers must vacate their spot if they catch fish with dolphin assistance while other fishers are waiting [39]. Some fishers work alone (keeping all catch), whereas others form long-term cooperative alliances by sharing spots, selling fish collectively and splitting profits [37]. The beach at the fishing site is a highly social place where fishers prepare nets, rest, define whose turn it is to go fishing, and interact with—and learn from—one another [37,38,41]. Fishers’ group composition changes throughout the day as fishers enter or exit the water (electronic supplementary material, figure S1), depending on conditions [37]—especially the presence of ‘good’ cooperative dolphins (known for efficient cooperation) [39,42]. Therefore, environmental and social factors dynamically alter fisher group composition throughout the day (electronic supplementary material, figure S1).

### (b) Data collection

We recorded behaviour, location, heart rate (HR) and heart rate variability (HRV) of 24 net-casting fishers at *Tesoura* beach, Laguna [41] (figure 1A). Data were collected between approximately 09.00 and 17.00 across 18 days over 2 years (eight in 2023; 10 in 2024), during the 3-week mullet migration peak (late May–early June) [36]. Ten fishers participated both years; eight only in 2023, and six only in 2024.

We used Polar Team Pro sensors (Polar Electro Oy, Finland) attached to two-electrode chest belts (figure 1C) to record electrocardiograms (ECG) at 1000 Hz and global positioning system (GPS) positions at 10 Hz. Up to 11 fishers were monitored simultaneously each day. As we were limited by the number of sensors, participants were selected based on their frequent presence at the site. All participants gave written informed consent. Fishers without sensors were included in group size measures but did not contribute to synchrony measurements.

Three trained observers conducted continuous all-event behavioural sampling [43], recording: (i) total number of fishers and dolphins present at the fishing site (figure 1B); (ii) confidential IDs of sensor-equipped fishers (figure 1C); (iii) timing of net-casts (figure 1D); (iv) whether casts followed dolphin cues; and (v) the outcome of net casts (figure 1E) quantified by the number of mullet caught (as in [37]). GPS data provided precise fisher positions and inter-individual distances during fishing (figure 1B). At the end of each sampling day, we conducted a brief, private interview using a semi-structured questionnaire (cf. [38]) to assess whether fishers (i) fished alone or cooperatively (with other fishers) that day, and (ii) if cooperatively, with whom (figure 1F). These data informed our analyses on the social context and cooperative bonds among fishers.

### (c) Data analysis

To examine physiological synchrony among fishers during collective fishing, we focused on events where fishers with HR sensors stood in the water together for at least 5 min (i.e. groups of 2−10 fishers; see electronic supplementary material, figure S1 for examples of the data). This threshold ensured sufficient duration for HRV synchrony to establish. To standardize across events, we restricted analyses to the first 20 min of observation windows. The time each group spent fishing together (summarized in 1 min bins) was included as a predictor in the analyses. HRV synchrony was then calculated for each dyad within each group using cross-wavelet power analysis (see below).

**Figure 2.**
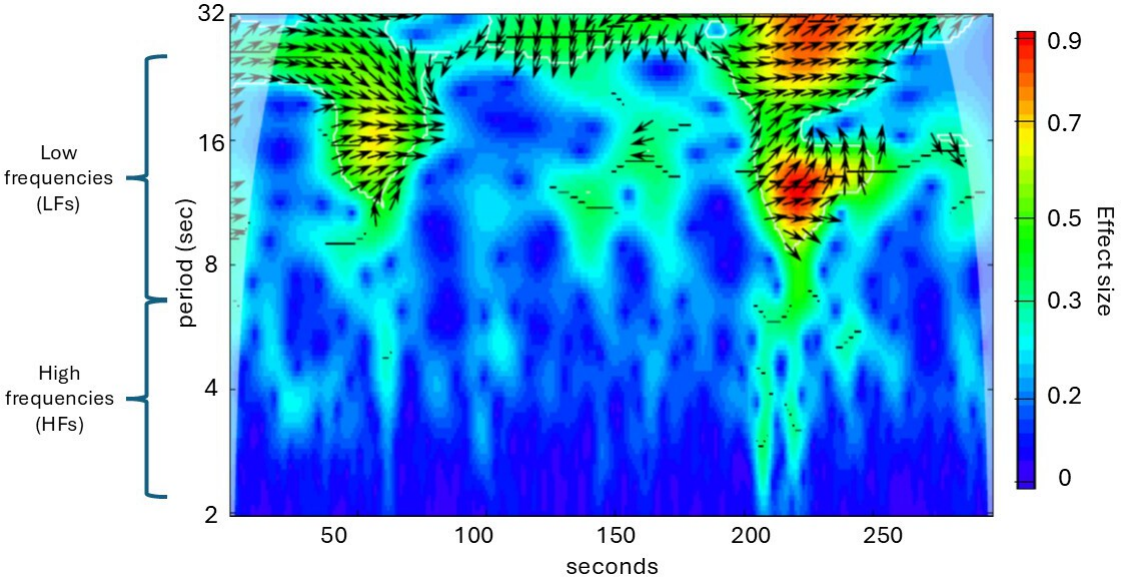
Cross-wavelet power plot of heart rate variability (HRV) synchrony in fisher dyads. Example plot showing HRV synchrony between two fishers over time (*x*-axis) and frequency bands (*y*-axis). Synchrony levels range from low (blue) to high (red). Lower frequencies (LFs), corresponding to longer HRV cycles, are shown on the upper *y*-axis (periods of 6.7 to 25 s [period = 1/frequency]); higher frequencies (HFs) appear lower on the *y*-axis (periods of 2.5 to 6.67 s). Arrows indicate the phase of HRV synchrony: right-pointing arrows represent in-phase synchrony, while left-pointing arrows indicate anti-phase synchrony.

We first converted ECG data to RR intervals (i.e. the time between successive heartbeats) using the native algorithm of the sensor manufacturer. Raw RR interval data were further processed in R 4.2.2 [44] using custom-made code [45] to remove artefacts and ectopic beats, and interpolate missing values. To align the raw data temporally across individuals, we created HR time series in beats per minute (bpm) with a resolution of 4 Hz. We then applied wavelet power analysis to obtain both time and frequency information by computing wavelet transforms of the HR time series. Pairwise synchronization of HR time series was assessed using cross-wavelet power analysis (R package WaveletComp; [46]). This method uses wavelet transformations—mathematically similar to Fourier transformations—to analyse signal power across different frequency bands. Specifically, it convolves the signal with scaled versions of a Morlet wavelet, enabling the assessment of frequency components with high temporal resolution [46,47]. Cross-wavelet power analysis then quantifies the covariance between wavelet-transformed HRV sequences across time and frequency bands (method reviewed in [27]) (figure 2), providing a dynamic measure of physiological synchrony between each fisher dyad.

Compared with traditional HRV analysis (LF and HF frequencies only), this approach of determining HRV synchrony (cross-wavelet power) provides finer time–frequency resolution across low and high frequency bands [48]. From the original cross-wavelet power spectrum bands ranging from 2 to 32 s per period (s/p), we extracted two frequency bands (figure 2) in a similar range as used in traditional fast Fourier Transform approaches (i.e. usually a lower HF band of 4−8 s/p , and an upper LF band of 8−16 s/p).

Alternative measures, like the cardiac sympathetic index (CSI: [49]) might allow for a cleaner distinction between purely sympathetic and parasympathetic influences, which the wavelet analytical approach, based on continuous HR data and depicting power across the high and low frequency bands, cannot provide. However, the CSI is derived from segments of data, e.g. 5-min periods, which would require alternative analytical approaches, e.g. cross-correlational methods.

### (d) Interpretation of cross-wavelet power analysis results

HRV synchrony values close to 1 indicate strong alignment of ANS activity between individuals; values near 0 indicate low alignment. This metric reflects the local covariance of two time series across time and frequency [48]. Power in low and high-frequency bands reflect different aspects of ANS regulation, offering unique insights into group physiology. Activity in LF bands is influenced by both sympathetic and parasympathetic systems, indicating shared arousal during stressful situations, physical activity and collective vigilance [50]. HR activity, driven by the vagus nerve via release of fast-acting acetylcholine, is linked to high frequency (HF) changes (>0.15 Hz), and reflects rapid adjustments during restful, socially engaging or cooperative states [51].

### (e) Statistical analysis

We fit two sets of models to investigate: (i) how social and environmental factors affect the HRV synchrony among fisher dyads, and (ii) whether HRV synchrony predicts group-level fishing success.

#### (i) Social and environmental factors affecting dyadic heart rate variability synchrony among fishers

To examine factors affecting HRV synchrony, we fit two Bayesian linear mixed models (one per frequency band) using a Markov chain Monte Carlo (MCMC) sampler in the *MCMCglmm* R package [52] to estimate model coefficients. This approach allowed us to use a multi-membership structure to account for both fisher identities within dyads and repeated measures across dyads and group compositions [38]. HRV synchrony coefficients in both LF and HF bands were log-transformed to meet normality assumptions and used as response variables. Eight fixed predictors were included in each model: duration of time fishers stood together in the water (5−20 min); square-rooted physical distance between fishers (mean = 16.0±16.8 m SD); total number of active fishers in the water (range = 1–26, mean = 12.7±5.2 SD, including fishers without HR sensors); whether any fisher in the dyad cast a net during that minute (*N*_yes_ = 2548, *N*_no_ = 37 124); whether fishers in the dyad cooperated with each other on that day (*N*_yes_ = 3891, *N*_no_ = 35 781); whether fishers in the dyad cooperated with any other fisher on that day (*N*_yes_ = 29 891, *N*_no_ = 9781); presence of dolphins at the fishing site (*N*_yes_ = 36 719, *N*_no_ = 2953); and whether a dolphin gave a foraging cue during the minute of the net-casting event (*N*_yes_ = 4277, *N*_no_ = 35 395). Random intercepts included fishers’ sensor IDs, date and a group ID.

All numeric predictors were scaled and centred at zero. Distances were square-root transformed to reflect the greater importance of small changes at close proximity. Since HRV synchrony was binned into 1 min intervals, other variables were averaged accordingly. Each MCMC chain ran for 300 000 iterations (burn-in = 100 000, thinning = 200 iterations). Among six prior structures tested (for the variance of R- and G-structures), the inverse Wishart distribution yielded best model diagnostics [52]. Predictors were considered significant if the 95% high posterior density (HPD) intervals excluded zero, indicating a robust directional effect.

#### (ii) Group-level heart rate variability synchrony and fishing success

To address how HRV synchrony was linked to group performance—measured as fishing success—we fit logistic regression models (one per frequency band) using the *lme4* R package [53]. These analyses focused on group-level data, using the HRV synchrony coefficients for each frequency band averaged across all dyads within a group for each minute. The response variable was binary: whether any group member caught fish during a given minute. The sole predictor was mean HRV synchrony (scaled and centred) averaged across all dyads per minute. Group identity and sampling day were included as random intercepts to account for the system’s intrinsic temporal variation. These analyses were restricted to minutes with at least one net cast, as fish capture was otherwise impossible. Even so, success was relatively rare—only 6.5% of minutes included a successful catch (*N*_yes_ = 43, *N*_no_ = 622), consistent with prior reports for this fishery [37]. As more fishers may enter the water during favourable fishing conditions, distances within dyads can increase, thereby reducing HRV synchrony (see §3). To account for this, we re-ran the fishing success models using only dyads that were closer than the overall median fisher–fisher distance (10.57  m).

## 3. Results

### (i) Social and environmental factors affecting dyadic heart rate variability synchrony among fishers

**Figure 3.**
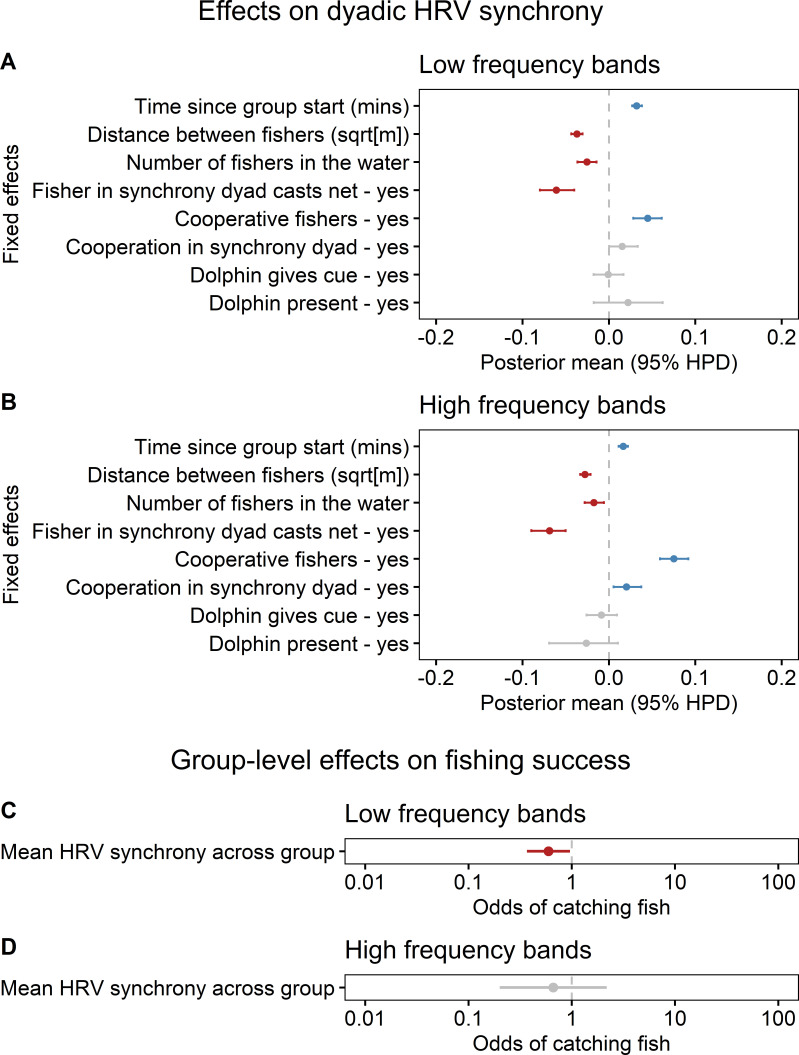
Influence of social and environmental factors on HRV synchrony and subsequent fishing success in artisanal fishers. (A,B) Results from Bayesian MCMC linear mixed models showing the effects of social and environmental predictors on HRV synchrony across two frequency bands of cross-wavelet power data: (A) LF and (B) HF bands. Points and whiskers represent posterior mean and 95% high posterior density (HPD) credible intervals of each predictor. These models assess how social and environmental predictors influence HRV synchrony across frequency domains. (C,D) Results from logistic regression models testing the effect of group-level HRV synchrony (scaled and centred) on fishing success (binary: yes/no) per minute: (C) LF synchrony, (D) HF synchrony. Points and whiskers represent estimated odds ratios with 95% confidence intervals on a log-scale. Effects are considered statistically significant when intervals (whiskers) do not overlap zero. Blue indicates positive influence, red negative and grey non-significant results.

We analysed HRV synchrony across 628 differently composed fisher groups, totalling 5011 min of observation. In both the LF and HF bands, HRV synchrony increased with the time fishers spent together in the water and decreased with greater physical distance between them (figure 3A,B). HRV synchrony also declined as the number of fishers in the water increased. The strongest disruptor of HRV synchrony was when a member of a dyad cast a net (figure 3A,B).

Long-term cooperative relationships among fishers were the strongest positive predictor of synchrony in both LF and HF frequency bands (figure 3A,B). General cooperation (i.e. with any other fisher) on that day had a stronger effect than cooperation within a specific dyad; the latter effect was only significant in the HF domain, which is related to social bonding and cooperation (figure 3B). Lastly, the dolphins’ presence or their foraging cues that trigger net-casting, had no significant effects on the fishers’ HRV synchrony in either LF or HF domains (figure 3A,B).

### (ii) Group-level heart rate variability synchrony and fishing success

HRV synchrony in the LF domain—linked to physical activity, collective arousal and shared alertness—was negatively associated with fishing success (figure 3C). HRV synchrony in the HF domain showed no significant effect on group-level hunting outcomes (figure 3D). When only considering the HRV synchrony among nearby fishers (i.e. those closer together than the median 10.57 m fisher–fisher distance), we again found a significant negative effect of synchrony in the LF band (mean LF synchrony ± SE=−0.52 ± 0.25, *p* = 0.03) and no significant effect of synchrony in the HF band (mean HF synchrony ± SE=−0.41 ± 0.61, *p* = 0.5, *n* = 594 groups) on the propensity to catch fish. This supports the notion that the negative association between LF synchrony and success is robust, and not an artefact of larger inter-fisher distances that can occur in bigger groups.

## 4. Discussion

Our investigation into the fine-scale physiological synchrony of humans during group hunting reveals that social factors drive synchronization of individuals’ autonomic nervous systems. Time spent together, closer physical proximity and cooperative strategies among artisanal fishers were positively associated with HRV synchrony, while physical distance and competitive dynamics were negatively associated with HRV synchrony. These results align with previous studies showing that individuals who are physically [54] or emotionally closer [55] tend to synchronize physiologically. In several taxa, proximity is known to enhance communication, social bonding and coordination (e.g. [56,57]), and our results corroborate that cooperation fosters further physiological alignment. In the context of the human–dolphin fishery, close physical proximity among fishers likely facilitates collective coordination and indicates social cohesion. Additional factors important for physiological synchronization, such as eye contact, vocal communication and behavioural matching, might also have occurred more frequently at close proximity.

HRV synchrony decreased when a fisher cast a net. This might reflect competitive tension among non-cooperative fishers disrupting group alignment, or could simply be the physiological impact from the physical activity of casting a net, which activates the sympathetic nervous system. Despite their central role in the success of this human–dolphin fishery [37,38], dolphin presence and activity had no significant effect on HRV synchrony. This suggests HRV synchrony is primarily driven by internal group dynamics, though it is also possible that fishers respond differentially depending on the identity of the dolphin (which we have not addressed here)—many can visually identify individual dolphins and distinguish which ones are most likely to help catch fish [39,42].

Interestingly, physiological synchrony increased even when only one member of a dyad cooperated with any other fisher, regardless of whether that specific cooperative behaviour was directed at their dyadic partner. This suggests a group-level effect of cooperation. In social foraging and ambush predation [34,58,59], synchronization—both behavioural and physiological—can enhance collective hunting efficiency. Group-hunting mammals [58] and some human hunter–gatherers [60,61] benefit from heightened coordination to maximize prey capture success. In this human–dolphin fishery, fishers’ coordinated responses to dolphin cues improve individual and group performance [37,62], particularly when fishers work in cooperative groups [38]. Given the speed and mobility of mullet schools, shared physiological arousal was hypothesized to enhance reaction time and group effectiveness.

Surprisingly, however, increased physiological synchrony was inversely associated with group hunting success. These findings possibly reflect the complexity of this social–ecological system, shaped by both cooperation and competition among the fishers [38,39,41], along with their mutualistic interactions with dolphins [36,37]. One explanation for the link between higher LF synchrony and reduced fishing success is that increased LF synchrony might reflect shared physiological arousal—such as heightened alertness or physical exertion—rather than effective coordination *per se*. In high-stakes, fast-paced environments like net casting assisted by dolphins, excessive arousal may reduce situational awareness and introduce noise into group decision-making. Moreover, effective fishing often requires temporally staggered actions to avoid net entanglement and respond flexibly to fish movement along the fisher line (electronic supplementary material, video S1). While physiological synchrony can facilitate alignment and responsiveness, it may also limit the adaptive variability necessary for efficient group performance. For instance, high HRV synchrony in a group task (volleyball team) was associated with lower performance [63]. These findings challenge the view that physiological synchrony is universally beneficial, revealing instead a context-dependent effect where excessive alignment may hinder collective success.

Our findings have broader implications beyond this human–wildlife mutualism. First, they highlight the complex role of physiological synchrony in shaping collective and cooperative behaviour. Collective physiology and its alignment is increasingly recognized as fundamental to group behaviour [12,22,24], decision-making [24,64] and cooperative success across social species [14,17,21,40,65]. We contribute to this recent body of knowledge by showing the impact of HRV components in real-time social and environmental contexts, revealing how coordination among foragers is deeply tied to their physiology. Second, our findings add new nuances to the literature suggesting that physiological synchrony may be a key underlying mechanism enabling humans to function as effective cooperative foragers. By demonstrating how different HRV components in humans respond to current social and environmental factors, we provide novel evidence that coordination and cooperation among foragers are tightly linked to their physiological states, with synchrony in physiological states also affected by spatial proximity. Future research should explore how this physiological synchrony evolves over time, the nuances and long-term consequences for foraging success, as well as potential parallels in other human–wildlife mutualistic systems [66], and whether different dimensions of synchrony (e.g. behavioural, hormonal) show similar dynamics in collective foraging.

## Data Availability

De-identified and anonymized data and R code used for the analyses is available from the OSF repository [67]. Supplementary material is available online [68].
